# DETERMINANTS OF AFFECT-BALANCE IN MONGOLIAN OLDER ADULTS

**DOI:** 10.1093/geroni/igae098.0137

**Published:** 2024-12-31

**Authors:** Saranchuluun Otgon, Denise Burnette, Zuchi Lkhamsuren, Fabio Casati, Yerkyebulan Mukhtar, Sugarmaa Myagmarjav

**Affiliations:** University of Trento, Trento, Trentino-Alto Adige, Italy; Virginia Commonwealth University, Richmond, Virginia, United States; Asia University, Taichung, Taichung, Taiwan (Republic of China); University of Trento, Trento, Trentino-Alto Adige, Italy; Mongolian National University of Medical Sciences, Ulaanbaatar, Ulaanbaatar, Mongolia; Mongolian National University of Medical Sciences, Ulaanbaatar, Ulaanbaatar, Mongolia

## Abstract

Objectives: In Mongolia, the number of persons aged ≥ 60 years is projected to increase from 125,000 (7%) in 2000 to more than 800,000 (25%) by 2050. Current policies and programs are inadequate to support integrated health services that ensure the physical, functional, and psycho-social components of healthy active aging. The purpose of this study was to assess levels and correlates of affect-balance, an indicator of life satisfaction and general well-being, in older adults in Mongolia. Methods: Data are from an in-person survey with urban and rural adults aged 55 and 88 (N=304). Affect-balance scores were derived from the Organization for Economic Cooperation Development (OECD) scale for measuring psychological well-being. Results: Both male and female study participants reported high (positive) affect-balance. Self-rated health, educational attainment, loneliness, and engagement in volunteer activities were significantly associated with affect-balance. Volunteering, a key component of ‘productive aging,’ notably contributed to high levels of positive emotions and low levels of negative emotions. Conclusion: Findings highlight the importance of fostering positive emotions in later life and can inform the development of targeted policies and interventions to enhance older Mongolians’ quality of life and well-being.

